# More than *DeQi*: Spatial Patterns of Acupuncture-Induced Bodily Sensations

**DOI:** 10.3389/fnins.2016.00462

**Published:** 2016-10-19

**Authors:** Won-Mo Jung, Woosun Shim, Taehyung Lee, Hi-Joon Park, Yeonhee Ryu, Florian Beissner, Younbyoung Chae

**Affiliations:** ^1^Acupuncture and Meridian Science Research Center, College of Korean Medicine, Kyung Hee UniversitySeoul, South Korea; ^2^Acupuncture, Moxibustion and Meridian Research Center, Division of Standard Research, Korea Institute of Oriental MedicineDaejeon, South Korea; ^3^Somatosensory and Autonomic Therapy Research, Institute for Neuroradiology, Hannover Medical SchoolHannover, Germany

**Keywords:** acupuncture, bodily sensation, *DeQi*, meridian, spatial information

## Abstract

Acupuncture uses needles to stimulate certain parts of the body, inducing a specific sensation, termed *DeQi*, which regard as essential for acupuncture's therapeutic effect. Here, we used the newly developed tool, bodily sensation mapping, to investigate the spatial configuration of acupuncture-induced sensations throughout the body. Twenty-five participants randomly received acupuncture stimulation or tactile stimulation using a von Frey filament at four different acupoints (HT7, PC6, ST36, and SP10) on the left side of the body. Subjects evaluated the characteristics of *DeQi* sensations and marked the areas of induced sensations on a body outline. We compared the psychophysical responses of *DeQi* sensations and visualized the spatial patterns of these sensations using statistical parametric mapping. We found greater intensity of *DeQi* sensations following acupuncture stimulation compared with tactile stimulation, with relatively small differences among the four acupoints. The sensation maps exhibited similar spatial patterns for acupuncture and tactile stimulation in the areas close to the stimulated sites. However, acupuncture was associated with additional sensations in areas remote from the stimulated sites. This study demonstrates that acupuncture stimulation produces greater *DeQi* sensations than tactile stimulation and results in the spreading of sensations to areas remote from the stimulus sites. Investigating the spatial patterns of acupuncture-induced sensations may be crucial for understanding the underlying mechanisms of acupuncture.

## Introduction

The theory of the so-called meridian system plays a central role in East-Asian medicine and therapeutic interventions such as acupuncture, moxibustion, cupping, and tuina. In this pre-scientific theory, *Qi* refers to a vital energy that was thought to circulate within meridians; the flow of this energy can be influenced in numerous ways. In this context, *DeQi* (Chinese for “obtaining the qi”) describes the unique sensations elicited by therapeutic interventions such as acupuncture (Zhou and Benharash, 2014). Achieving the appropriate *DeQi* sensation is known to play an important role in acupuncture analgesia (Kong et al., 2005; Choi et al., 2013). *DeQi* has been described as a combination of various sensations, including heaviness, numbness, soreness, distension, and warmth, and may have affective connotations, such as feeling refreshed or relieved (Kim et al., 2008; Yuan et al., 2013). It is generally accepted that the main manifestation of *DeQi* sensations should be separate from the acute pain at the site of the needling, especially in the case of sharp pain (MacPherson and Asghar, 2006). Many neuroimaging studies suggest that *DeQi* is associated with specific brain activation patterns and may play a crucial role in the efficacy of acupuncture (Hui et al., 2005, 2007; Napadow et al., 2009; Asghar et al., 2010; Beissner et al., 2012; Chae, 2012; Wang et al., 2013).

Acupuncture is defined as the insertion of needles into the body at specific points, and the sensations produced can be local or remote from the site of stimulation (Zhu et al., 1981; Chae et al., 2013). A number of acupuncture sensation questionnaires focus on the assessment of sensory qualities and the intensity of sensation during acupuncture stimulation (Kong et al., 2007; White et al., 2008). In contrast, little work has been done to explore the spatial patterns of bodily sensations following acupuncture stimulation. Recently, Beissner and Marzolff (2012) used a geographic information system and proposed that laser acupuncture evoked propagating sensations along the meridian in close connection with classical meridian pathways (Beissner and Marzolff, 2012). Accumulated clinical knowledge of acupuncture has given rise to the assumption that the spatial properties of acupuncture-induced sensations may be closely related to the therapeutic outcomes of acupuncture treatments (Lee et al., 2013; Jung et al., 2015). Considering the aforementioned properties of acupuncture stimulation, it would be useful to assess the spatial configuration of acupuncture-induced sensations throughout the body.

In this study, we evaluated the characteristics of the *DeQi* responses to acupuncture or tactile stimulation at four points (HT7, PC6, ST36, and SP10) and investigated the spatial configuration of acupuncture-induced sensations across the body using bodily sensation maps (BSMs). We hypothesized that the sensation patterns of the acupuncture stimuli would be more extended far from the stimulated site compared to those of the tactile stimuli.

## Materials and methods

### Subjects

Healthy participants were recruited via posters. They had no known history of neurological, psychiatric, or visual disorders. Participants were not allowed to drink alcohol or caffeine or take any drugs or medications on the day of the experiment. They were informed of the nature of the experiment and provided written informed consent. None of the participants had any prior knowledge of acupuncture or meridian theory. The study was conducted in accordance with the Declaration of Helsinki and approved by the Institutional Review Board of Kyung Hee University.

### Experimental design and procedure

The experiments were carried out in an air-conditioned room (24 ± 2°C). We conducted the entire experiment in the same season, i.e., winter season. In order to minimize the non-specific feeling from the cold weather to air-conditioned room, the participants were asked to take some rest for at least 30 min to be accustomed to the temperature-controlled room. Prior to commencing the experiments, basic demographic and background data were collected from all participants using the Acupuncture Expectancy Scale (AES) and Acupuncture Fear Scale (AFS) (Kim et al., 2013, 2014).

All participants were asked to change into comfortable clothes and to lie down in a comfortable position on an acupuncture table for all sessions. They received alternating acupuncture and tactile stimulation at two different points over four sessions and were asked to evaluate their psychophysical responses in terms of the *DeQi* scale and the spatial configuration of the bodily sensations that arose in response to each stimulus. Subjects were randomly assigned to one of two groups according to whether they first received the arm session (A-L) or the leg session (L-A). They received acupuncture stimuli at two points (HT7 or PC6 in the arm and ST36 or SP10 in the leg). The stimulus type (i.e., acupuncture or tactile stimulation) was applied in pseudorandom order.

The whole experiment was composed of one baseline measurement session and eight experiment sessions. Prior to commencing the stimulation sessions, the participants were asked to focus on sensations in their whole body for 60 s as part of a baseline measurement. They were instructed to report the location of any bodily sensations that arose during the 60 s period. The subjects' eyes were covered using a blindfold, and ear plugs were used to minimize auditory interruptions. After removing the blindfold, subjects were given 105 s to evaluate their psychophysical responses.

**Figure 1:**
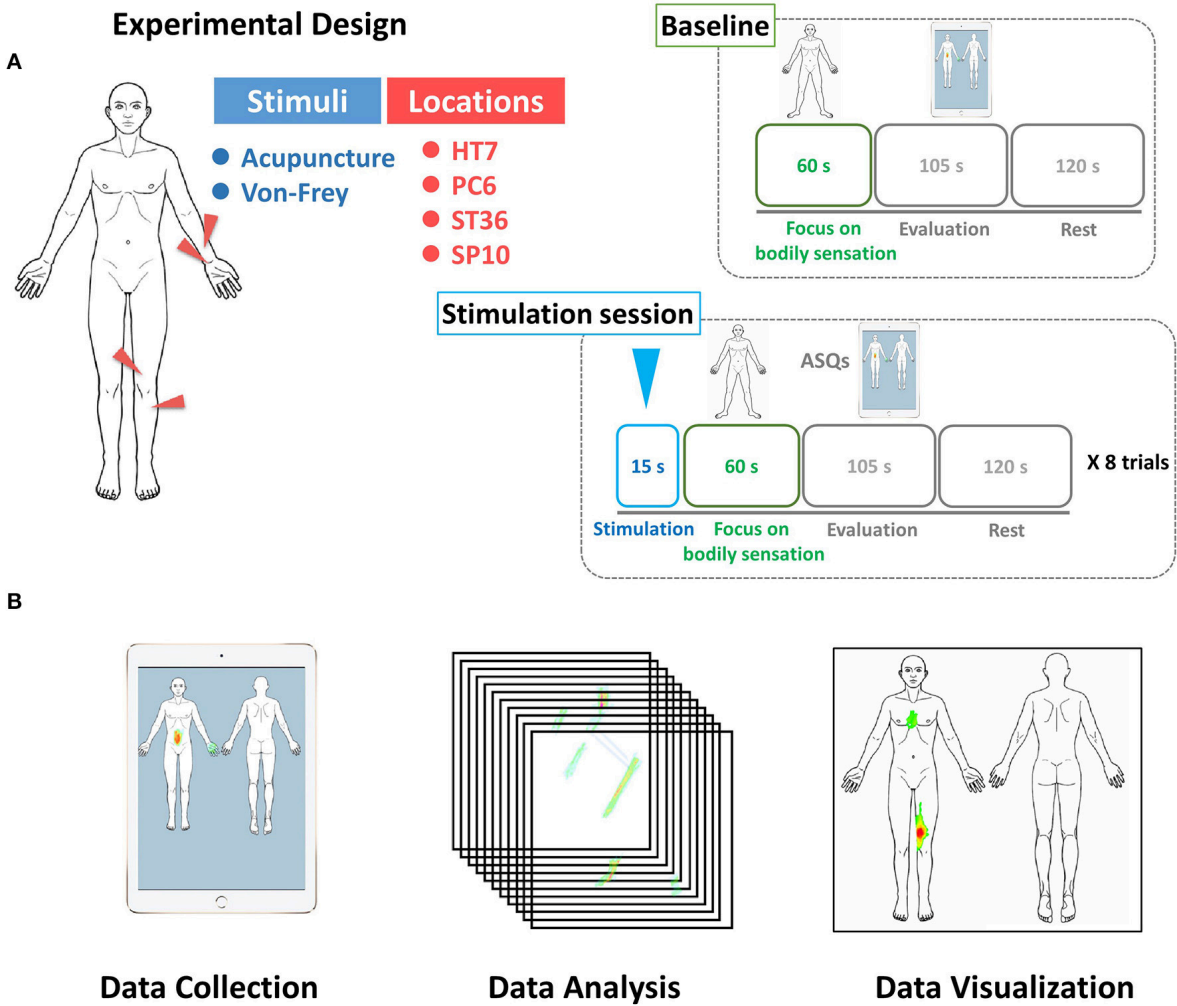
**(A)** Experimental design. All participants received acupuncture or tactile stimulation at four different points (HT7, PC6, ST36, and SP10) on their left side. In each session, they were asked to evaluate the characteristics of *DeQi* sensations from the body and to mark the areas of the acupuncture-induced sensations using the BSM device. **(B)** Participants were asked to draw their bodily sensations using the BSM system. The BSM system was used to gather spatial information on the acupuncture sensations (Data collection). We calculated the statistical significance of associations between each condition and the spatial configuration of the acupuncture-induced sensations (Data analysis). We visualized the spatial distribution of acupuncture-induced sensations using the human body template by applying Z scores (Data visualization).

Each experimental session lasted for 5 min, during which the subjects received either acupuncture or tactile stimulation for 15 s and were then asked to focus on the location of the sensations in their body for 60 s. The subjects' eyes were covered using a blindfold, and ear plugs were used to minimize auditory interruptions. After removing the blindfold, subjects were given 105 s to evaluate their psychophysical responses. Finally, the subjects could relax for a period of 120 s prior to the subsequent session (Figure 1A).

### Acupuncture and tactile stimulation

All participants received acupuncture stimulation at two different points on the left arm (HT7 and PC6) and two different points on the left leg (ST36 and SP10). HT7 is located on the anteromedial aspect of the wrist, radial to the flexor carpi ulnaris tendon, on the palmar wrist crease. PC6 is located on the anterior aspect of the forearm, between the tendons of the palmaris longus and the flexor carpi radialis, 2 B cun proximal to the palmar wrist crease. ST36 is located on the anterior aspect of the leg, on the connecting ST35 with ST41, 3B cun inferior to ST35 (ST35 is located on the anterior aspect of the knee, in the depression lateral to the patellar ligament and ST41 is located on the anterior aspect of the ankle, in the depression at the center of the front surface of the ankle joint, between the tendons of extensor halluces longus and extensor digitorium longus). SP10 is located on the anteromedial aspect of the thigh, on the bulge of the vastus medialis muscle, 2 B cun superior to the medial end of the base of the patella. Two acupoints in the arm and two acupoints the leg were included in this experiment because they belong to the two different meridians, but within a similar anatomical region. The needles for acupuncture stimulation were 0.25 mm in diameter and 40 mm in length (Dong Bang, Gyeonggi-do, Korea), and they were inserted to a depth of 10 mm. The manipulation technique was insertion, followed by rotation in alternating directions at 1 Hz for 15 s, followed by immediate withdrawal.

Tactile stimulation was delivered at the same sites using a von Frey filament (North Coast Medical, San Jose, CA) with a mass of 15 g with a frequency of 1 Hz. Care was taken to ensure that both stimulation methods were as similar as possible, except that the tactile stimulation did not penetrate the skin but instead poked on the surface at the same site. To control for expectation effects, all participants were told that they would be stimulated by acupuncture only. We used von Frey filament to control for both tactile stimulation of cutaneous somatosensory receptors over the acupoint, as well as the cognitive processing induced by participants expecting acupuncture stimulation (Napadow et al., 2009). Tactile stimulation was simulated by poking the skin over the acupoints with von Frey filament. To avoid possible infections or other side effects, the practitioner sanitized hands using an alcohol-based hand sanitizer before acupuncture or tactile stimulations.

### Psychophysical measurement of *DeQi* sensations

Subjects were asked to evaluate the psychophysical characteristics of *DeQi* sensations using the Acupuncture Sensation Questionnaire (ASQ) (Kim et al., 2008). This questionnaire was developed based on the sensations that patients experienced during actual acupuncture sessions. It includes not only the traditional *DeQi* sensations (e.g., dull, numb, and heavy) around the needling sites but also global changes in bodily sensations (e.g., feelings of refreshment and relief).

As we were interested in the changes in sensation across the body, participants were asked to evaluate these sensations on scale ranging from 0 (nothing) to 3 (extreme) following each session. The following ASQ descriptors were used in our study: Q1, dull; Q2, numb; Q3, spreading; Q4, warm; Q5, relief of tense or tight muscles; Q6, gentle (soft) touch; Q7, heavy; Q8, compressing or pressing; Q9, refreshing or relieving; Q10, activated digestion with bowel motion; Q11, streaming opening flow of a previously stuffed or blocked feeling; Q12, activated circulation; and Q13, pricking.

### Acquisition of data on the spatial configuration of bodily sensations

Following the focus on the body and subsequent stimulation, participants were asked to report the locations of their bodily sensations using the BSM, which presents a template of the human body as 2D images, with in front and back views (Download this image and the program from the website: http://cmslab.khu.ac.kr/downloads/bsm). The user can change the color of the points on a continuous color map via successive strokes on a region with a touch pen (Wacom Inc, OR, USA). The resulting drawings were collected in 1024 × 1024 matrices using an iPad (Apple Inc. Cupertino, CA, USA; Figure 1B).

### Data analysis

Statistical analysis of the *DeQi* sensation data was performed using the R software package (http://cran.r-project.org). To compare the intensities of the *DeQi* sensations for each question over two different stimulation types (i.e., to determine the effect of stimulus type) and four different stimulus locations (i.e., to determine the effect of stimulus location), a two-way analysis of variance (ANOVA) was used.

To represent the spatial patterns of the sensations under each condition, we extracted the parametric maps of bodily sensations for the four stimulus locations and two stimulation types (i.e., acupuncture and von Frey filament) using the BSM tool. Individual datasets for each session were normalized within the range 0–1. Two approaches were used to derive group-level sensation maps. With the first, the normalized drawings were subjected to group-level analysis. With the second, an additional step, whereby we subtracted the baseline sensation map from the maps obtained following the stimulation session, was introduced.

The group-level analysis consisted of an analysis of random effects using a pixel-wise univariate t-test on individual sensation maps within a mask of the body template (3dttest, AFNI, http://afni.nimh.nih.gov/afni). Additionally, paired t-test was applied between bodily sensation patterns of the eight experimental sessions and those of the baseline measurement session. Statistical analysis on spatially distributed multiple data might involve multiple comparison problem. In order to obtain corrected type I error of < 0.05 for the statistical analysis in the whole body, 10,000 Monte Carlo simulations with AFNI AlphaSim package could calculate a compromised cluster size of >1089 pixels under individual p-value < 0.05 (Ledberg et al., 1998). In the statistical maps, statistical t-values were transformed into Z-scores of the pixels which reflect significant spatial information on bodily sensations. The color code was visualized according to Z-score.

## Results

### Baseline characteristics

Twenty-five right-handed participants (age 24.4 ± 0.6 years; 13 females) took part in the study. The AES score was 11.1 ± 0.6, and the AFS score was 31.8 ± 2.1.

### Psychophysical responses to *DeQi* sensations

**Figure 2:**
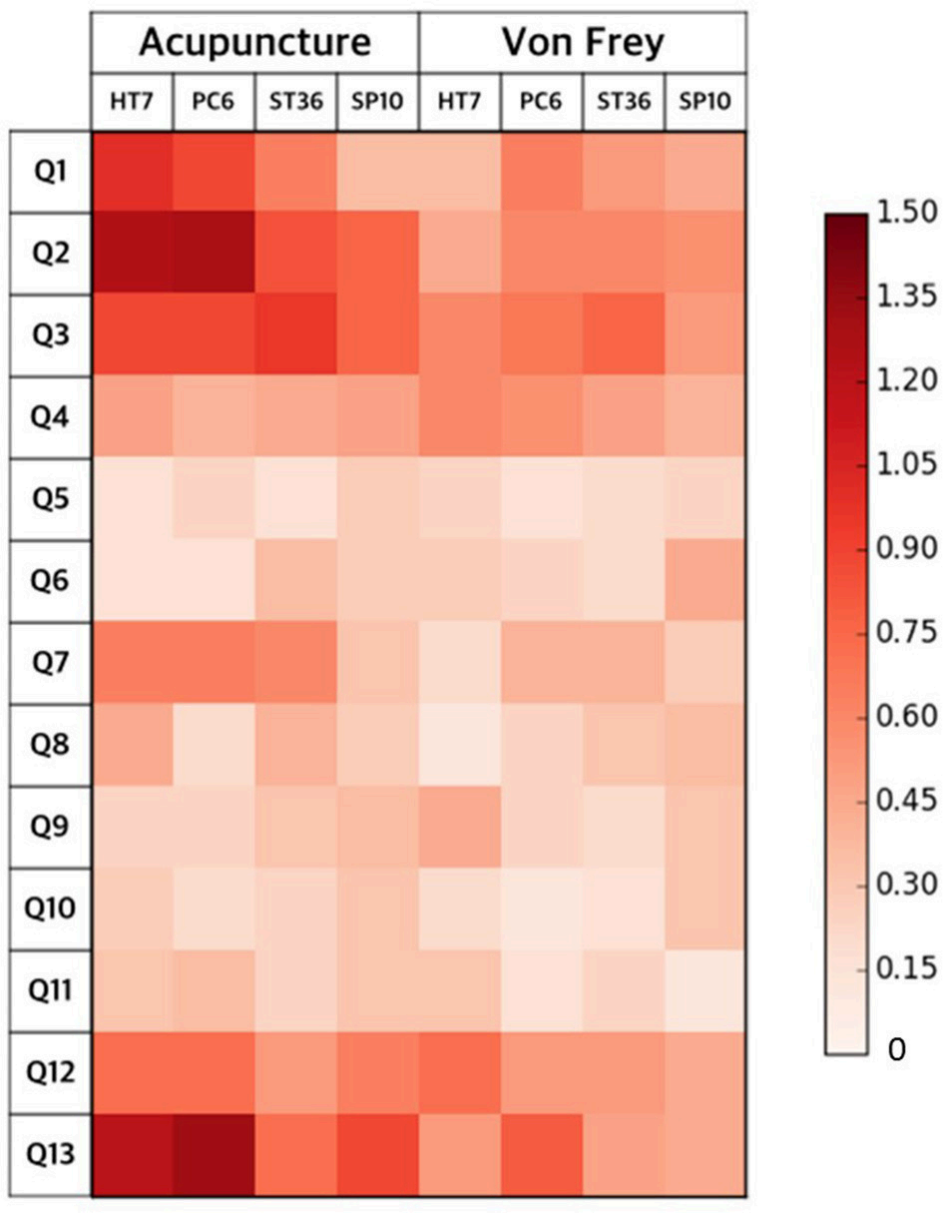
*****DeQi*** intensities of the two types of stimulation at four acupoints**. The single items were Q1, dull; Q2, numb; Q3, spreading; Q4, warm; Q5, relief of tense or tight muscles; Q6, gentle (soft) touch; Q7, heavy; Q8, compressing or pressing; Q9, refreshing or relieving; Q10, activated digestion with bowel motion; Q11, streaming opening flow of a previously stuffed or blocked feeling; Q12, activated circulation; and Q13, pricking. Overall, we found that the *DeQi* sensations following acupuncture stimulation were more intense than those following tactile stimulation, with small differences among the four different acupoints.

We visualized the *DeQi* intensity for the two different types of stimulus and the four different acupoints using a color-coded matrix (Figure 2). Table 1 lists the results of the individual ANOVA tests. Visual inspection revealed greater intensity of *DeQi* sensations for acupuncture compared with tactile stimulation, whereas differences among the four different acupoints were small. A two-way ANOVA revealed significant *stimulus effects* for Q2 (numb), Q3 (spreading out), Q7 (heavy), and Q13 (pricking), as well as significant *location effects* for Q13 (see Table 1). No interaction effects (i.e., stimulus × location) were observed.

**Table 1:** **Psychophysical responses to ***DeQi*** sensations**.

	**Acupuncture**				**Tactile**				**Stimuli effect**		**Locations effect**		**Interact**	
	**HT7**	**PC6**	**ST36**	**SP10**	**HT7**	**PC6**	**ST36**	**SP10**	***F***	***P***	***F***	***P***	***F***	***P***
Q1	1.0 ± 0.2	0.9 ± 0.2	0.6 ± 0.1	0.4 ± 0.2	0.4 ± 0.1	0.6 ± 0.2	0.5 ± 0.1	0.4 ± 0.2	3.68	0.06	1.68	0.17	1.60	0.19
Q2^#^	1.2 ± 0.2	1.3 ± 0.2	0.8 ± 0.2	0.8 ± 0.2	0.4 ± 0.1	0.6 ± 0.2	0.6 ± 0.2	0.6 ± 0.2	15.59	0.0001	1.06	0.37	1.57	0.20
Q3^*^	0.9 ± 0.2	0.9 ± 0.2	1.0 ± 0.1	0.8 ± 0.2	0.6 ± 0.2	0.7 ± 0.2	0.8 ± 0.2	0.5 ± 0.1	3.76	0.05	0.60	0.62	0.03	0.99
Qi4	0.5 ± 0.1	0.4 ± 0.1	0.4 ± 0.2	0.5 ± 0.1	0.6 ± 0.2	0.6 ± 0.1	0.5 ± 0.1	0.4 ± 0.1	0.34	0.56	0.18	0.91	0.27	0.85
Q5	0.2 ± 0.1	0.2 ± 0.1	0.2 ± 0.1	0.3 ± 0.1	0.2 ± 0.1	0.2 ± 0.1	0.2 ± 0.1	0.2 ± 0.1	0.00	1.00	0.21	0.89	0.23	0.87
Q6	0.2 ± 0.1	0.2 ± 0.1	0.4 ± 0.2	0.3 ± 0.1	0.3 ± 0.1	0.2 ± 0.1	0.2 ± 0.1	0.4 ± 0.1	0.39	0.53	0.81	0.49	0.81	0.49
Q7^*^	0.6 ± 0.2	0.6 ± 0.2	0.6 ± 0.1	0.3 ± 0.2	0.2 ± 0.1	0.4 ± 0.1	0.4 ± 0.2	0.3 ± 0.2	4.53	0.03	0.85	0.47	0.58	0.63
Q8	0.4 ± 0.2	0.2±0.1	0.4 ± 0.1	0.3 ± 0.2	0.1 ± 0.1	0.2 ± 0.1	0.3 ± 0.1	0.4 ± 0.2	0.51	0.47	0.37	0.77	0.85	0.47
Q9	0.2 ± 0.1	0.2 ± 0.1	0.3 ± 0.1	0.4 ± 0.1	0.4 ± 0.1	0.2 ± 0.1	0.2 ± 0.1	0.3 ± 0.1	0.02	0.90	0.47	0.70	0.79	0.50
Q10	0.3 ± 0.1	0.2 ± 0.1	0.2 ± 0.1	0.3 ± 0.1	0.2 ± 0.1	0.1 ± 0.1	0.2 ± 0.1	0.3 ± 0.1	0.69	0.41	0.89	0.45	0.08	0.97
Q11	0.3 ± 0.1	0.4 ± 0.1	0.2 ± 0.1	0.3±0.1	0.3 ± 0.1	0.2 ± 0.1	0.2 ± 0.1	0.1 ± 0.1	1.74	0.19	0.33	0.81	0.58	0.63
Q12	0.7 ± 0.2	0.7 ± 0.2	0.5 ± 0.2	0.6 ± 0.2	0.7 ± 0.2	0.5 ± 0.2	0.5 ± 0.1	0.4 ± 0.2	0.72	0.40	0.59	0.62	0.24	0.87
Q13^#^	1.2 ± 0.2	1.3 ± 0.2	0.7 ± 0.1	0.9 ± 0.2	0.5 ± 0.1	0.8 ± 0.2	0.5 ± 0.1	0.4 ± 0.1	20.05	0.00001	3.96	0.009	0.76	0.52

*Q1, dull; Q2, numb; Q3, spreading; Q4, warm; Q5, relief of tense or tight muscles; Q6, gentle (soft) touch; Q7, heavy; Q8, compressing or pressing; Q9, refreshing or relieving; Q10, activated digestion with bowel motion; Q11, streaming opening flow of a previously stuffed or blocked feeling; Q12, activated circulation; and Q13, pricking. Data are given as mean ± standard error*.

# *Bonferroni-corrected*.

* *p < 0.05, uncorrected*.

### Spatial configuration of acupuncture-induced sensations

**Figure 3:**
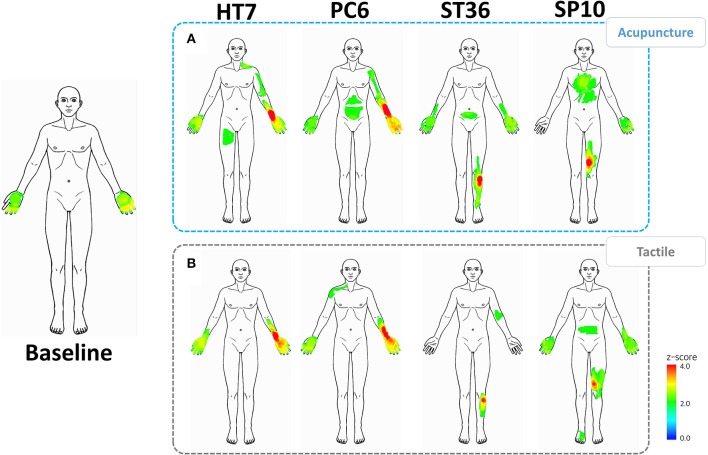
**Baseline bodily sensation patterns and spatial patterns of the acupuncture-induced sensations**. The left column shows the baseline spatial patterns. **(A)** spatial patterns following acupuncture stimulation at the HT7, PC6, ST36, and SP10 acupoints. **(B)** Spatial patterns following tactile stimulation at the HT7, PC6, ST36, and SP10 acupoints.

Bodily sensation patterns following acupuncture and tactile stimulation were visualized for each of the four acupoints (Figure 3). For comparison, baseline ratings are also shown.

Common spatial patterns were found in the bilateral palms at baseline as well as following the stimulation sessions. We further observed significant sensations in areas remote from the stimulus sites in the acupuncture sessions. For example, acupuncture stimulation at HT7 and PC6 resulted in sensations in the upper medial part of the arm, corresponding to propagating sensations along the meridian. Acupuncture stimulation at acupoint ST36 resulted in sensations in the abdomen, whereas SP10 stimulation resulted in sensations in the chest. Tactile stimulation at these acupoints also resulted in remote sensations, but there were relatively localized around the point of stimulation.

**Figure 4:**
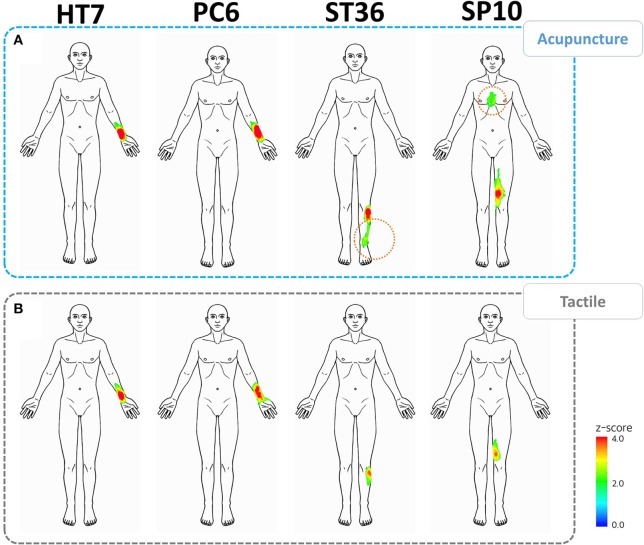
**Spatial patterns of acupuncture-induced sensations corrected for baseline bodily sensations**. **(A)** Spatial patterns following acupuncture stimulation at the HT7, PC6, ST36, and SP10 acupoints. **(B)** Spatial patterns following tactile stimulation at the HT7, PC6, ST36, and SP10 acupoints. Acupuncture stimulations, especially at ST36 and SP10, were associated with larger spatial patterns, which spread farther from the stimulus sites (dotted orange lines).

Following baseline correction, the spatial patterns following acupuncture and tactile stimulation increased in similarity and became more localized around the stimulus sites (Figure 4). However, we still found that sensations occurred in areas remote from the stimulus sites for the acupoints on the leg. These remote sensations were observed for acupuncture but not for von Frey stimulation. For acupoint ST36, referral of sensation was toward the lower part of the leg, corresponding to a propagating sensation, whereas, for acupoint SP10, referred sensations occurred in the upper part of the leg and the chest.

## Discussion

We have shown that acupuncture stimulation produced stronger *DeQi* sensations than tactile stimulation. We found that differences of the *DeQi* sensations among four different acupoints were small. Acupuncture stimulation resulted in more widespread spatial patterns of sensation than tactile stimulation and included areas remote from the stimulus site. This study represents the first statistical analysis of acupuncture-induced sensations using visualizations of spatial patterns using a human body template.

We found significant differences in the intensity of *DeQi* sensations between acupuncture stimulation and tactile stimulus in terms of sensations described as “numb,” “spreading out,” “heavy,” and “pricking.” These sensations are the main manifestations of *DeQi*, and are generally considered to differ from the acute pain at the needling site (MacPherson and Asghar, 2006). It is believed that *DeQi* sensations are central to the therapeutic effect of acupuncture. Only one questionnaire item (i.e., “pricking”) reflected a significant difference in intensity among the different locations (HT7, PC6, ST36, and SP10). It has been reported that *DeQi* sensations exhibit significant variation depending on the anatomical location of the acupoints, even along a given meridian (Yin et al., 2009). The different intensities of pricking sensations among different acupoints in our study may be a result of the different distributions of nociceptors or different sensory transmission systems according to the different anatomical structures.

One distinctive feature of *DeQi* sensations involves spread or radiation from the stimulus sites. We found that the sensation patterns for the acupuncture stimulus extended far from the stimulated site, whereas those for the tactile stimulation were limited to the region of the stimulus site. Acupoints of the arm were associated with patterns of sensation referral that differed from those of the leg. Whereas acupuncture stimulation at acupoints HT7 and PC6 resulted in the spreading of sensations to the upper medial part of the arm, stimulation at acupoints ST36 and SP10 caused referred sensations in the abdomen and the chest. The spatial configuration of acupuncture stimulation at ST36 resulted in line-like sensations along the lower part of the leg, which is similar to the course of the stomach meridian. These phenomena are so-called propagating sensations along the channel (PSC), a common phenomenon reported in a number of Chinese studies conducted in the late 1970s (Li, 1980; Ji, 1981) as well as more recently (Park et al., 2002; Hui et al., 2011). According to the early studies, ~20% of patients receiving acupuncture reported PSC. In contrast, Beissner and Marzolff reported a much larger figure, 82.4%, under conditions of sensory deprivation (Beissner and Marzolff, 2012). In terms of methodology, participants were asked to sketch the human body on the illustrations, and the sensation patterns were visualized by overlapping sensation polygons (Beissner and Marzolff, 2012). In comparison to the previous study, we applied a newly developed tool, or the BSMs based on iPad, and therefore could acquire the data of bodily sensations in a more direct way. We could visualize the spatial patterns of acupuncture-induced sensations using statistical parametric mapping methods.

The bilateral palm areas were the sites of sensations in the baseline as well as in almost all stimulus sessions. To investigate stimulation-specific spatial patterns, it is necessary to exclude such non-specific bodily sensations, which may arise when subjects focus on their body. However, following baseline correction, acupuncture stimulation at acupoints ST36 and SP10 resulted in significant spatial patterns in areas remote from the stimulus sites. Such sensations were reported in the chest area for SP10 (*Z* score = 2.438), which is far from the stimulus site. Notably, these referred sensations were predominantly proximal to the site of stimulation. Taking into account classical acupuncture practices, the locations of target diseases for acupuncture treatment appear to be associated with the route of meridian system (Jung et al., 2015). It appears that these spatial sensation patterns may be related to the target organs for acupuncture treatment along the classical route of the Spleen meridian, which begins on the medial tip of the big toe and ascends through the diagram, over the chest. However, further investigation of clinical and experimental data is needed to explore associations between acupuncture-induced sensations and the routes of meridians. With larger sample sizes and the enrollment of relevant patients, we believe that the BSM and similar tools will offer a promising approach with which to investigate PSC phenomena and the remote sensation effects of acupuncture.

Still, our study possesses several limitations. First, 2-min washout period might not be enough time to eliminate the sensory from the previous stimuli. Even though two acupoints and the stimuli types were applied in pseudorandom order in the current study, we cannot fully exclude the possible interactions of acupuncture and tactile stimulation, or stimulation from different points. Second, as we only included four different acupoints in the current study, we were not able to show spatial patterns of bodily sensations to non-acupoints at this stage. In order to deal with the point-specificity issue, it would be interesting to compare spatial patterns of bodily sensations between acupoints and non-acupoints in the future study. Third, since each participant evaluated bodily sensation to each stimulation only once, we were not able to calculate the variabilities within the subjects in the current study. Future studies are needed to investigate the intra-rater and/or interrater variability.

In summary, we found that acupuncture stimulation resulted in greater *DeQi* sensations and was associated with spatial patterns that extended farther from the stimulus site than tactile stimulation. Investigation of the associations between acupuncture-induced sensations and the corresponding meridian system could improve our understanding of the mechanisms underlying acupuncture treatment.

## Author contributions

Conceived and designed the experiments: WJ, WS, YC. Performed the trial: WS, TL. Analyzed the data: WJ, YC. Discussed the data: WJ, WS, TL, HP, YR, FB, YC. Wrote the first draft of the paper: WJ, YC. Revised the paper and approved the final version: WJ, WS, TL, HP, YR, FB, YC.

### Conflict of interest statement

The authors declare that the research was conducted in the absence of any commercial or financial relationships that could be construed as a potential conflict of interest.
